# Anesthetic Management of Intravenous Leiomyomatosis With Intracardiac Extension Using Preemptive Cardiopulmonary Bypass: A Case Report

**DOI:** 10.7759/cureus.83435

**Published:** 2025-05-03

**Authors:** Koichiro Seki, Reiko Yamamoto, Koichi Yoshinaga, Mamoru Takeuchi

**Affiliations:** 1 Anesthesiology and Critical Care Medicine, Jichi Medical University, Shimotsuke, JPN

**Keywords:** anesthetic challenges, cardiopulmonary bypass, intracardiac extension, intravenous leiomyomatosis, right heart failure

## Abstract

Intravenous leiomyomatosis (IVL) with intracardiac extension can cause circulatory collapse during anesthetic induction due to right heart obstruction. We report the case of a 63-year-old woman with IVL extending into the right ventricle, presenting with right heart failure and shock. To maintain hemodynamic stability and facilitate tumor resection, we established cardiopulmonary bypass (CPB) under local anesthesia before inducing general anesthesia. Preoperative imaging revealed a tumor extending from the right ovarian vein to the right ventricle, causing circulatory failure. In the operating room, CPB was initiated via femoral cannulation under local anesthesia with analgosedation to maintain spontaneous breathing, followed by general anesthesia induction. A median sternotomy was performed, and an additional venous cannula was placed in the superior vena cava to achieve total CPB. The tumor was resected from the right heart and inferior vena cava. The patient was weaned from CPB and ventilation without complications. Pathology was later confirmed to be IVL. She was discharged on postoperative day 30. Establishing CPB before anesthetic induction maintained hemodynamic stability in this patient with IVL, intracardiac extension, and right heart failure, allowing for safe tumor resection.

## Introduction

Intravenous leiomyomatosis (IVL) is a rare smooth muscle cell tumor arising from either the uterine venous system or the uterine leiomyoma. It can extend into the inferior vena cava (IVC) and even into the right heart chambers, known as intracardiac extension of leiomyomatosis. IVL is often diagnosed incidentally. Resection is the only treatment of choice, but the incidental nature of the disease allows little time for careful planning of surgery and anesthesia. Another hemodynamic concern in managing this disease is the possibility of incarceration in any part of the right heart circulation from the IVC to the pulmonary artery, which can lead to circulatory collapse in the resection process [1]. Intravenous leiomyomatosis (IVL) with cardiac extension poses significant challenges for anesthetic management, particularly in the presence of right heart failure. Conventional anesthetic induction techniques using intravenous agents can lead to severe hemodynamic instability and cardiovascular collapse in these patients [2]. However, literature regarding the optimal anesthetic approach for managing IVL with right heart dysfunction remains scarce. We report a case in which we successfully maintained hemodynamic stability throughout the critical induction period, allowing for the safe removal of the obstructing intracardiac tumor by initiating cardiopulmonary bypass (CPB) under local anesthesia with analgosedation before induction of general anesthesia.

## Case presentation

A 63-year-old female (height 144.7 cm, weight 62.7 kg) with a history of hypertension and hyperlipidemia, treated with oral medications, presented to our hospital. She had a surgical history of total abdominal hysterectomy and bilateral salpingo-oophorectomy for uterine myoma and ovarian tumor 11 years prior. Two days before admission, she suffered from flu-like symptoms and lower leg edema, accompanied by repeated vomiting. On the day of admission, she visited a local hospital where her blood pressure was unmeasurable, and her oxygen saturation (SpO_2_) had decreased to 85%.

**Figure 1:**
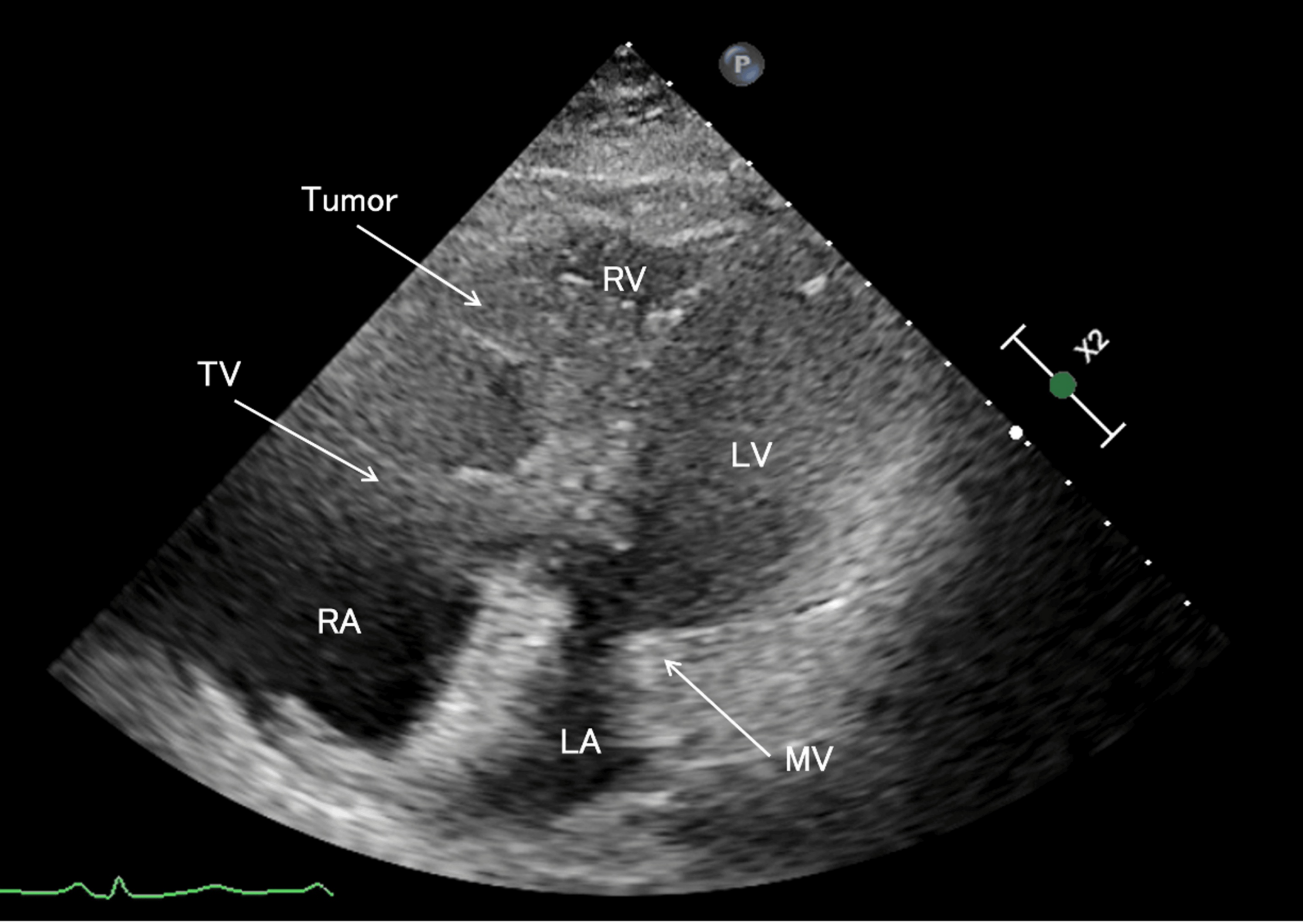
Transthoracic echocardiography, four-chamber view upon arrival A giant tumor originating from the inferior vena cava is extended into the right ventricle, becoming entrapped in the tricuspid valve during diastole. A mass was observed moving between the right atrium and right ventricle in synchrony with cardiac contractions across the tricuspid valve. The right ventricular contractility appeared visually preserved, but the left ventricle demonstrated a D-shaped deformity. The mass was partially cystic and appeared as a spherical, isoechoic structure. TV, tricuspid valve; RA, right atrium; RV, right ventricle; MV, mitral valve; LA, left atrium; LV, left ventricle

She was subsequently transferred to our hospital for emergency care. Upon arrival, her consciousness was alert. Her vital signs were as follows: temperature 35.9°C, heart rate 84 bpm, blood pressure 103/69 mmHg, and SpO_2_ 68% on 2 L/minute oxygen. The electrocardiogram showed a heart rate of 82 bpm and a right bundle branch block. A transthoracic echocardiography (TTE) revealed an ejection fraction of 61.7%, with a left ventricular end-diastolic diameter of 32.9 mm and a left ventricular end-systolic diameter of 21.9 mm. The left atrium measured 45.2 mm × 53.2 mm, and the right atrium measured 42.0 mm × 45.4 mm. The E/A ratio was 0.76. A pericardial effusion of up to 6 mm was observed anterior to the right ventricle. Trivial tricuspid regurgitation (TR) was noted; however, a mass was observed moving between the right atrium and right ventricle in synchrony with cardiac contractions across the tricuspid valve, which precluded accurate measurement of tricuspid inflow velocity. No other significant valvular abnormalities were detected. The contractility of the right ventricle appeared visually preserved, but the left ventricle demonstrated a D-shaped deformity. The mass measured 50 mm × 60 mm, was partially cystic, and appeared as a spherical, isoechoic structure. The IVC was filled with the tumor (Figure 1).

Laboratory investigations revealed a hematocrit of 51.4%, indicating hemoconcentration and polycythemic tendency, with a decreased platelet count of 98×10^3^/μL. Coagulation studies demonstrated coagulation abnormalities with PT-INR (prothrombin time and international normalized ratio) of 2.23 and APTT (activated partial thromboplastin time) of 40.5 seconds. Biochemical analysis showed evidence of hepatic dysfunction with AST 431 U/L, ALT 434 U/L, and γGTP 287 U/L, as well as renal impairment with BUN 63 mg/dL and creatinine 3.26 mg/dL. Arterial blood gas analysis, performed while the patient was receiving oxygen at 5 L/minute, demonstrated pH 7.421, pCO_2_ 24.6 mmHg, pO_2_ 135.0 mmHg, HCO_3_^-^ 15.7 mmol/L, and lactate 5.9 mmol/L, consistent with lactic acidosis due to circulatory failure (Table 1).

**Table 1:** The laboratory findings upon patient arrival The arterial blood gas analysis results are under 5 L/minute oxygen inhalation.

Complete Blood Count	Laboratory Values	Reference Range
White Blood Cell	19.4 × 10^3^/µL	3.3-8.6 × 10^3^/µL
Red Blood Cell	5.63 × 10^6^/µL	3.86-4.92 × 10^6^/µL
Hemoglobin	16.6 g/dL	11.6-14.8 g/dL
Hematocrit	51.4%	35.1-44.4%
Platelet	98 × 10^3^/µL	158-348 × 10^3^/µL
Coagulation	-	-
Prothrombin Time International Normalized Ratio	2.23	0.85-1.15
Activated Partial Thromboplastin Time	40.5 seconds	22.4-37.4 seconds
D-Dimer	14.8 µg/mL	<1 µg/mL
Biochemistry	-	-
C-Reactive Protein	0.95 mg/dL	0.00-0.14 mg/dL
Total Protein	5.9 g/dL	6.6-8.1 g/dL
Albumin	3.7 g/dL	4.1-5.1 g/dL
Aspartate Transaminase	431 U/L	13-30 U/L
Alanine Transaminase	434 U/L	7-23 U/L
Lactate Dehydrogenase	1117 U/L	124-222 U/L
Alkaline Phosphatase	207 U/L	38-113 U/L
Gamma-Glutamyl Transpeptidase	287 U/L	9-32 U/L
Creatine Kinase	2241 U/L	41-153 U/L
Creatine Kinase MB	136 U/L	≦12 U/L
Blood Urea Nitrogen	63 mg/dL	8-20 mg/dL
Creatinine	3.26 mg/dL	0.46-0.79 mg/dL
Sodium	134 mmol/L	138-145 mmol/L
Potassium	3.6 mmol/L	3.6-4.8 mmol/L
Chloride	87 mmol/L	101-108 mmol/L
Calcium	8.6 mg/dl	8.8-10.1 mg/dL
Inorganic Phosphorus	7.8 mg/dL	2.7-4.6 mg/dL
Glucose	161 mg/dL	73-109 mg/dL
Seroimmunoserological test	-	-
N-Terminal pro Brain Natriuretic Peptide	3261.0 pg/mL	<165 pg/mL
Troponin T	0.143 ng/mL	<0.014 ng/mL
Arterial blood gas analysis (Under 5 L/min Oxygen Inhalation)	-	-
pH	7.421	-
pCO₂	24.6 mmHg	-
pO₂	135.0 mmHg	-
HCO₃⁻	15.7 mmol/L	-
Base Excess	-6.4 mmol/L	-
Lactate	5.9 mmol/L	-

**Figure 2:**
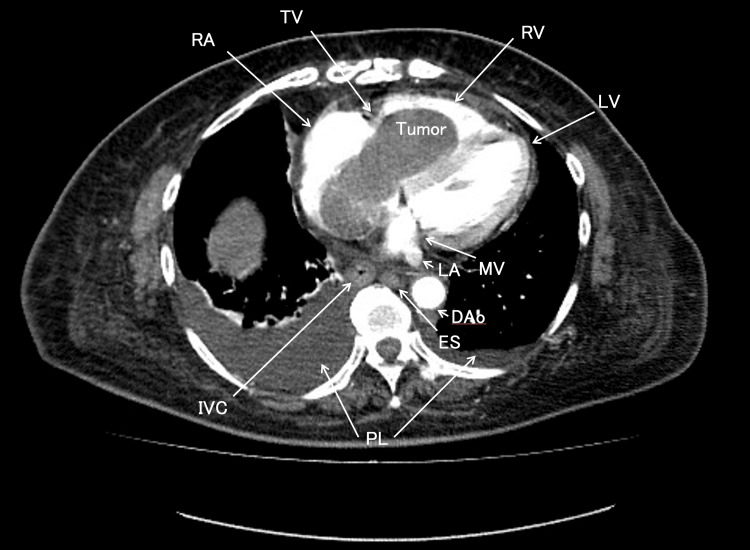
The axial plane of contrast-enhanced early-phase chest computed tomography A giant tumor is entrapped in the tricuspid valve and extends to the right ventricle. A bilateral pleural effusion was observed with right-sided predominance, and the inferior vena cava was filled with tumor. TV, tricuspid valve; RA, right atrium; RV, right ventricle; MV, mitral valve; LA, left atrium; LV, left ventricle; IVC, inferior vena cava; PL, pleural effusion; ES, esophagus; DAo, descending aorta

**Figure 3:**
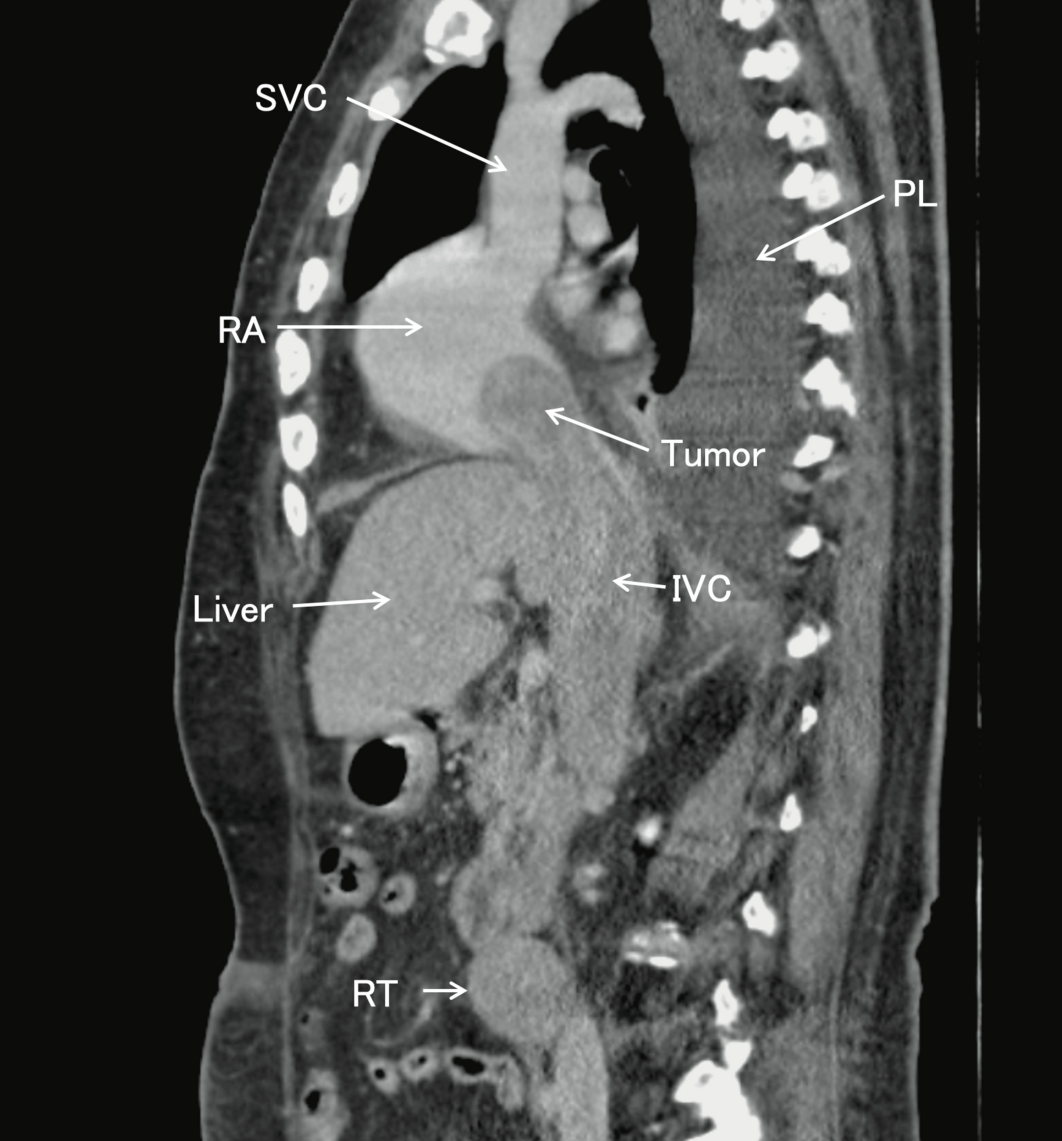
The sagittal plane of contrast-enhanced delayed phase chest and abdominal computed tomography The site of tumor recurrence is found in the retroperitoneum, invading the inferior vena cava and extending into the cardiac cavity. The inferior vena cava is filled with tumors. Pleural effusion is observed in the right thoracic cavity. SVC, superior vena cava; RA, right atrium; PL, pleural effusion; IVC, inferior vena cava; RT, site of tumor recurrence

Contrast-enhanced computed tomography (CT) showed a tumor extending from the right ovarian vein to the right ventricle, strongly suggesting intravenous leiomyomatosis (Figures 2, 3). Other CT findings included right kidney atrophy, fatty liver, and right-sided dominant pleural effusion. No active inflammatory findings or tumorous lesions were observed in the lung fields. No thrombi were detected in the deep veins of the lower extremities, and no pulmonary embolism was observed. No abnormalities were found in the abdominal organs, and no significant enlargement of the mediastinal, hilar, supraclavicular, abdominal, or pelvic lymph nodes was noted.

The patient was diagnosed with right heart failure and circulatory failure caused by a right-sided cardiac tumor obstructing pulmonary blood flow. She was admitted to the intensive care unit (ICU) for hemodynamic stabilization and initial resuscitation. An arterial line in the right radial artery and a central venous catheter in the right internal jugular vein were inserted in the ICU. However, her blood pressure continued to decline, leading to obstructive shock. Upon clinical deterioration, her vital signs showed significant hypotension with a blood pressure of 66/47 mmHg, heart rate of 83 beats per minute, and respiratory rate of 27 breaths per minute, requiring norepinephrine at 0.1 µg/kg/minute. Although her SpO_2_ reached 100% with 5 L/minute oxygen, she had strong respiratory effort and a respiratory rate of 27 breaths per minute. Emergency surgery was planned to stabilize her hemodynamics and remove the tumor from the right heart and the IVC as much as possible through median sternotomy. Considering her obstructive shock state and the strong respiratory effort with a rapid breathing pattern and use of accessory muscles, we determined that rapid induction of general anesthesia with conventional positive pressure ventilation carried a high risk of circulatory collapse. Therefore, for safety, we planned to establish CPB under local anesthesia with analgosedation before inducing general anesthesia. Furthermore, as total CPB was necessary for tumor removal from the right heart, with the possibility of requiring circulatory arrest and the need for additional venous cannulation of the superior vena cava (SVC) during surgery, we planned to establish CPB rather than venoarterial extracorporeal membrane oxygenation (VA-ECMO).

Following her transfer to the operating room and the placement of monitors, the patient received whole-body disinfection, draping, and preoxygenation. In the operating room, her initial blood pressure was 138/72 mmHg with norepinephrine at 0.1 µg/kg/minute, heart rate was 75 bpm, oxygen saturation was 100% with 5 L/minute oxygen, respiratory rate was 25 breaths per minute, and central venous pressure (CVP) ranged from 25 to 31 mmHg, with significant respiratory variations in arterial blood pressure and CVP. Midazolam 1 mg and fentanyl 50 µg were carefully administered repeatedly while closely monitoring the patient's spontaneous breathing and blood pressure. Under local anesthesia and analgosedation, the right femoral artery and vein were exposed, and a 16 Fr arterial cannula and a 24 Fr venous cannula were inserted. The venous cannula was placed at a depth of 25 cm, referring to the preoperative CT, at the level of the ovarian vein before reaching the tumor. CPB was initiated after administering 20,000 units of heparin and confirming an activated clotting time (ACT) of 767 seconds. As a flow rate of 2.5 L/minute was achieved, rapid induction was performed with midazolam, fentanyl, and rocuronium, and endotracheal intubation was performed under stable hemodynamics. Anesthesia was maintained with propofol, remifentanil, and rocuronium. The hemodynamics were maintained without increasing or adding inotropic and vasoconstrictor agents. After securing the airway with endotracheal intubation, a transesophageal echocardiography (TEE) probe was inserted, and the surgery began.

**Figure 4:**
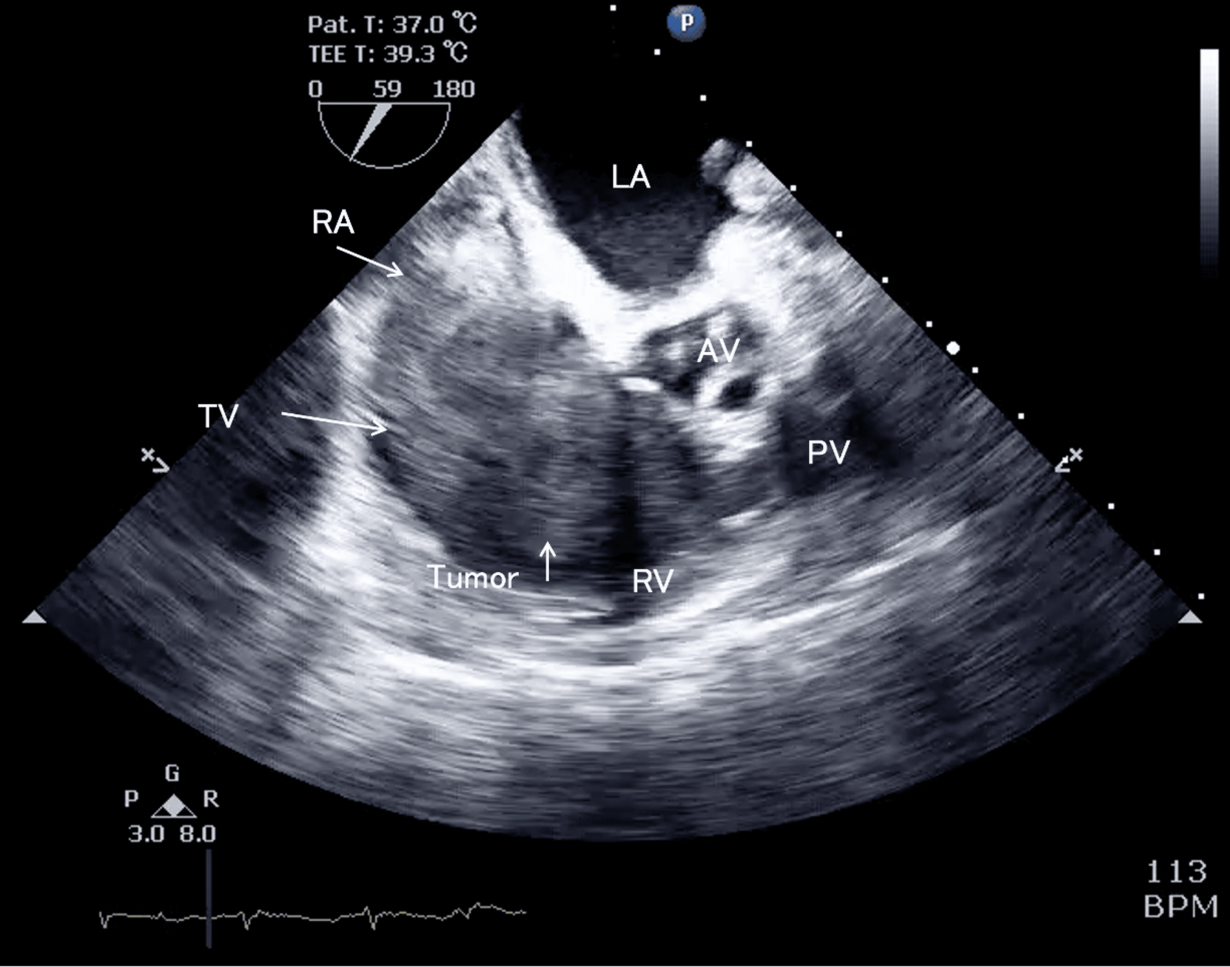
Intraoperative transesophageal echocardiography, mid-esophageal right ventricle inflow-outflow view As in Figure 1, the tumor was large enough to occupy the right atrium, with a spherical protrusion extending into the cardiac chamber that moved to and fro in synchrony with the cardiac cycle. During diastole, the tumor was entrapped in the tricuspid valve. Although the right ventricle was dilated, the right ventricular function was preserved. TV, tricuspid valve; RA, right atrium; RV, right ventricle; MV, mitral valve; LA, left atrium; LV, left ventricle; AV, aortic valve; PV, pulmonary valve

**Figure 5:**
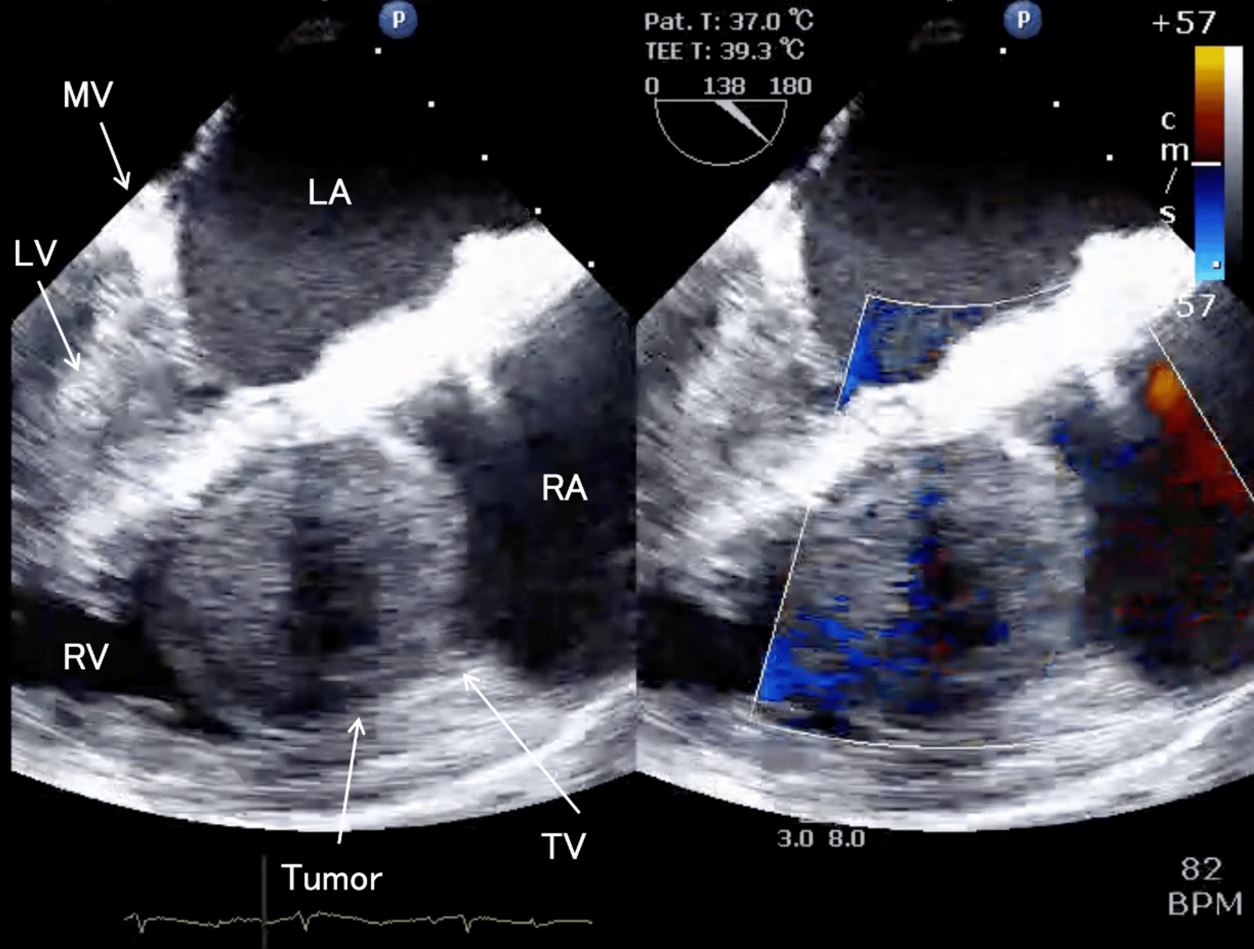
Intraoperative transesophageal echocardiography, color comparing mode of rotated mid-esophageal modified four-chamber view As in Figure 4, a giant tumor is entrapped in the tricuspid valve and obstructs right ventricular inflow. TV, tricuspid valve; RA, right atrium; RV, right ventricle; MV, mitral valve; LA, left atrium; LV, left ventricle

**Figure 6:**
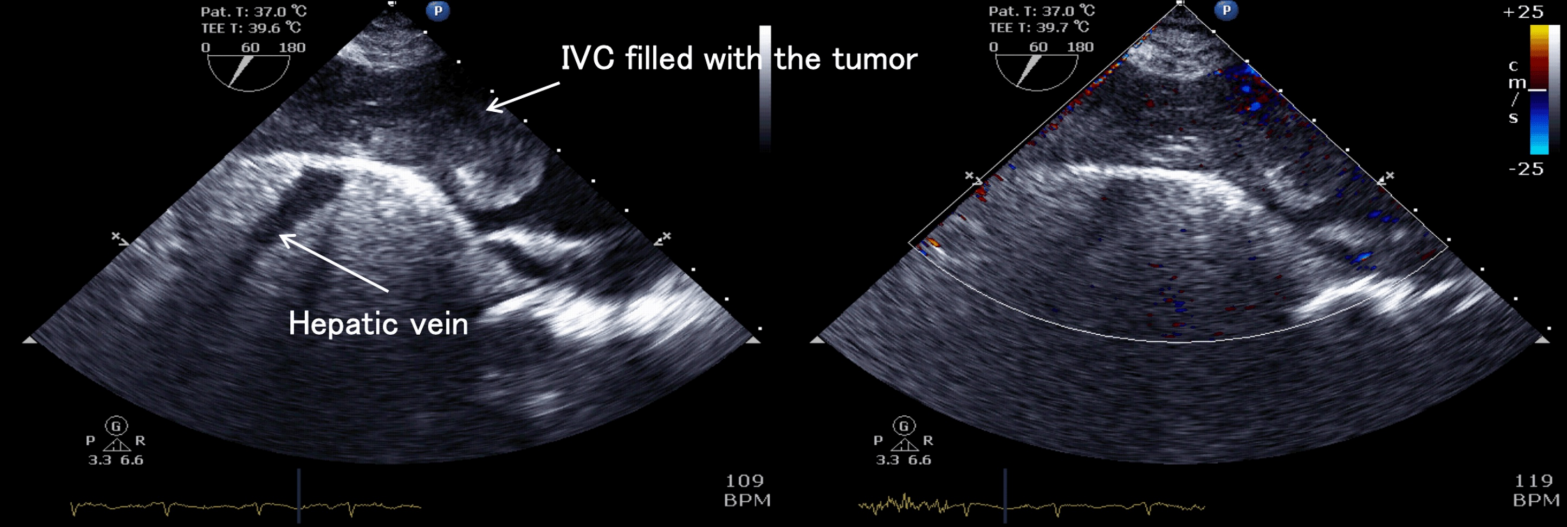
Intraoperative transesophageal echocardiography, transgastric inferior vena cava/hepatic veins view with color Doppler mode The inferior vena cava was filled with tumors, and blood flow in the inferior vena cava could barely be detected even when lowering the velocity scale. The tumor appeared to be fixed to the inferior vena cava wall without movement. The leading edge of the tumor extended to the right atrium, and the portion of the tumor within the right atrium showed to-and-fro mobility in synchrony with the cardiac cycle. No tumor was observed within the hepatic veins. IVC, inferior vena cava

Despite being obtained after the establishment of CPB, the TEE findings did not significantly differ from the TTE results. The left ventricular ejection fraction was 65%, the left ventricular end-diastolic diameter measured 25.5 mm, and the right ventricular end-diastolic diameter was 32.4 mm. The left ventricle exhibited a D-shaped deformity. The tricuspid annular plane systolic excursion was 18.5 mm. The interatrial septum was deviated toward the left atrium; however, no interatrial communication was observed. Pericardial effusion was present anterior to the right ventricle, measuring a maximum of 13.7 mm. Apart from trivial TR, no significant valvular abnormalities were identified. The tumor appeared attached to the IVC, measuring 66 mm × 45 mm, and presented as a spherical mass protruding into the right atrium; however, the tumor did not adhere to the cardiac cavity. The tumor demonstrated to-and-fro motion synchronously with the cardiac cycle and entrapping in the tricuspid valve during diastole, occupying a substantial portion of the right atrium. Significant pleural effusion was observed in the right thoracic cavity. No embolic findings were seen in the visible pulmonary arteries. Although no tumor invasion was observed in the hepatic veins, the visible IVC was almost filled with the tumor, with only minimal blood flow detected by color Doppler mode (Figures 4-6). The TEE findings visually demonstrated right heart underfilling.

**Figure 7:**
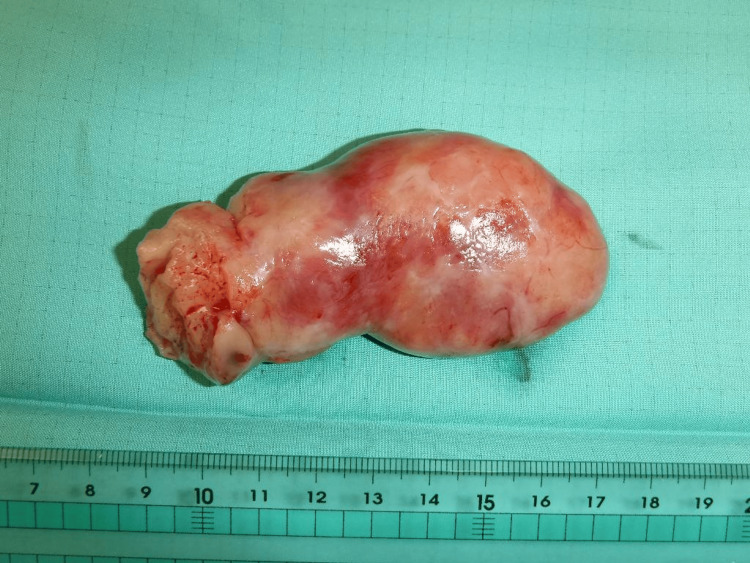
The specimen removed from the cardiac cavity The left side of the figure is the inferior vena cava side. The specimen's surface is smooth, and a notch is seen where it was fitted into the tricuspid valve.

A median sternotomy was performed, a 21 Fr arterial cannula was inserted into the ascending aorta, and a 32 Fr venous cannula was additionally inserted into the SVC to achieve full flow. A left atrial vent was inserted through the right superior pulmonary vein, and a cardioplegia cannula was inserted into the ascending aorta. The aorta was cross-clamped, and cardiac arrest was achieved with antegrade cardioplegia. The SVC was taped and snared, and the right atrium was opened. The IVC had minimal blood flow, providing an excellent surgical field. The tumor extended from the IVC into the cardiac cavity, passing through the tricuspid valve and invading the right ventricle. Still, it was not adhered to the surrounding tissues and was easily removed. The tumor removal from the IVC was limited to the visible range from the right atrium (Figure 7). After a successful resection, adequate blood flow from the IVC was observed. The IVC was taped and snared, and the right atrium was closed.

There were no complications, such as an airlock, and CPB operated stably throughout the procedure. After releasing the aortic cross-clamp, the heart spontaneously resumed beating, and CPB was easily separated with temporary atrial pacing at 80 bpm and dobutamine at 4.0 µg/kg/minute. Immediately after separation, oxygenation was adequate, with pH 7.363, pO_2_ 314 mmHg, pCO_2_ 36.2, and HCO_3_^-^ 20.1 on 100% inhaled oxygen.

**Figure 8:**
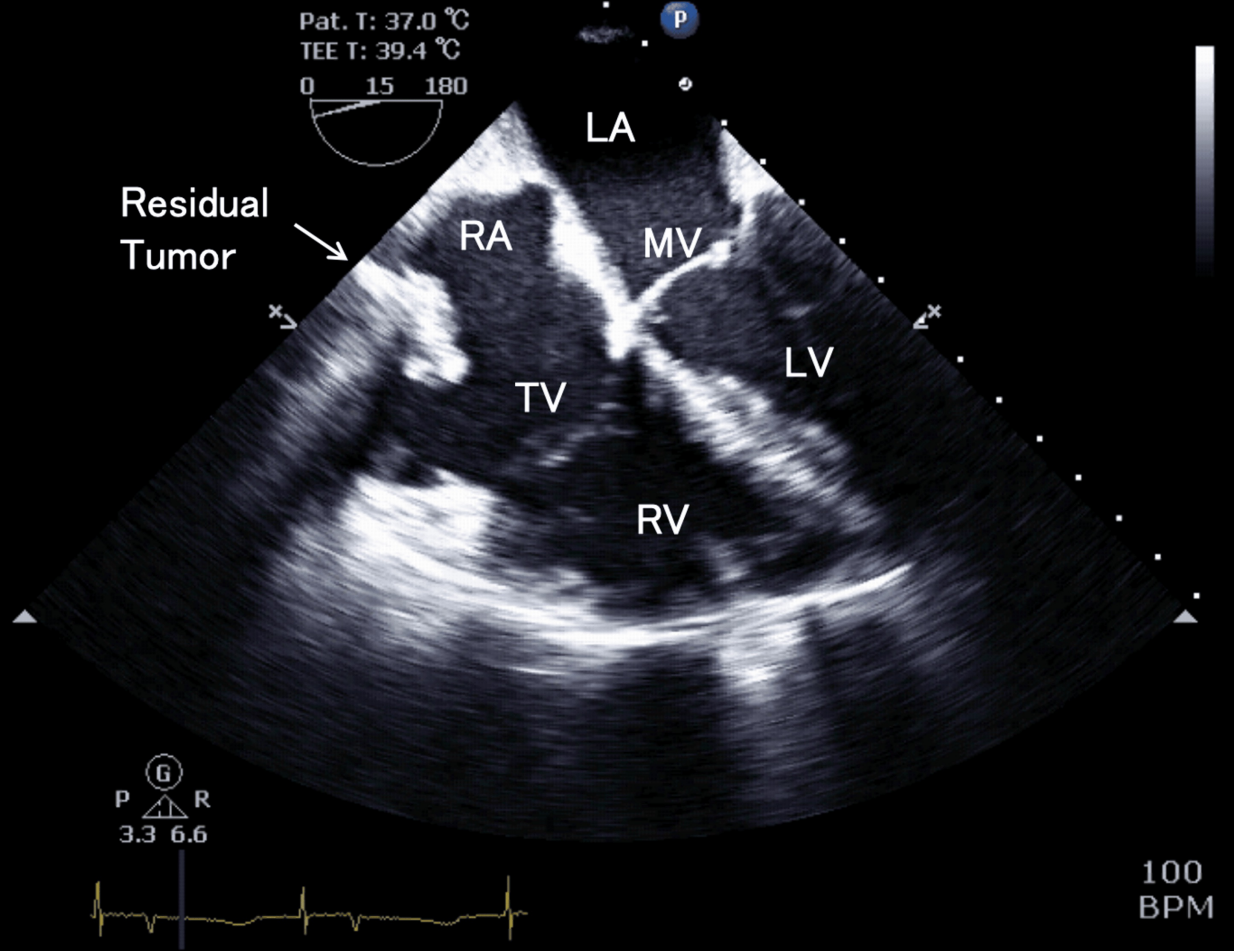
Post-procedural transesophageal echocardiography, four-chamber view This is a four-chamber view image taken after tumor resection and weaning from cardiopulmonary bypass. Although the intracardiac tumor was resected as much as possible, the image shows a residual tumor extending into the inferior vena cava. The right ventricular function was preserved, while the right ventricular cavity remained more prominent than the left. TV, tricuspid valve; RA, right atrium; RV, right ventricle; MV, mitral valve; LA, left atrium; LV, left ventricle

**Figure 9:**
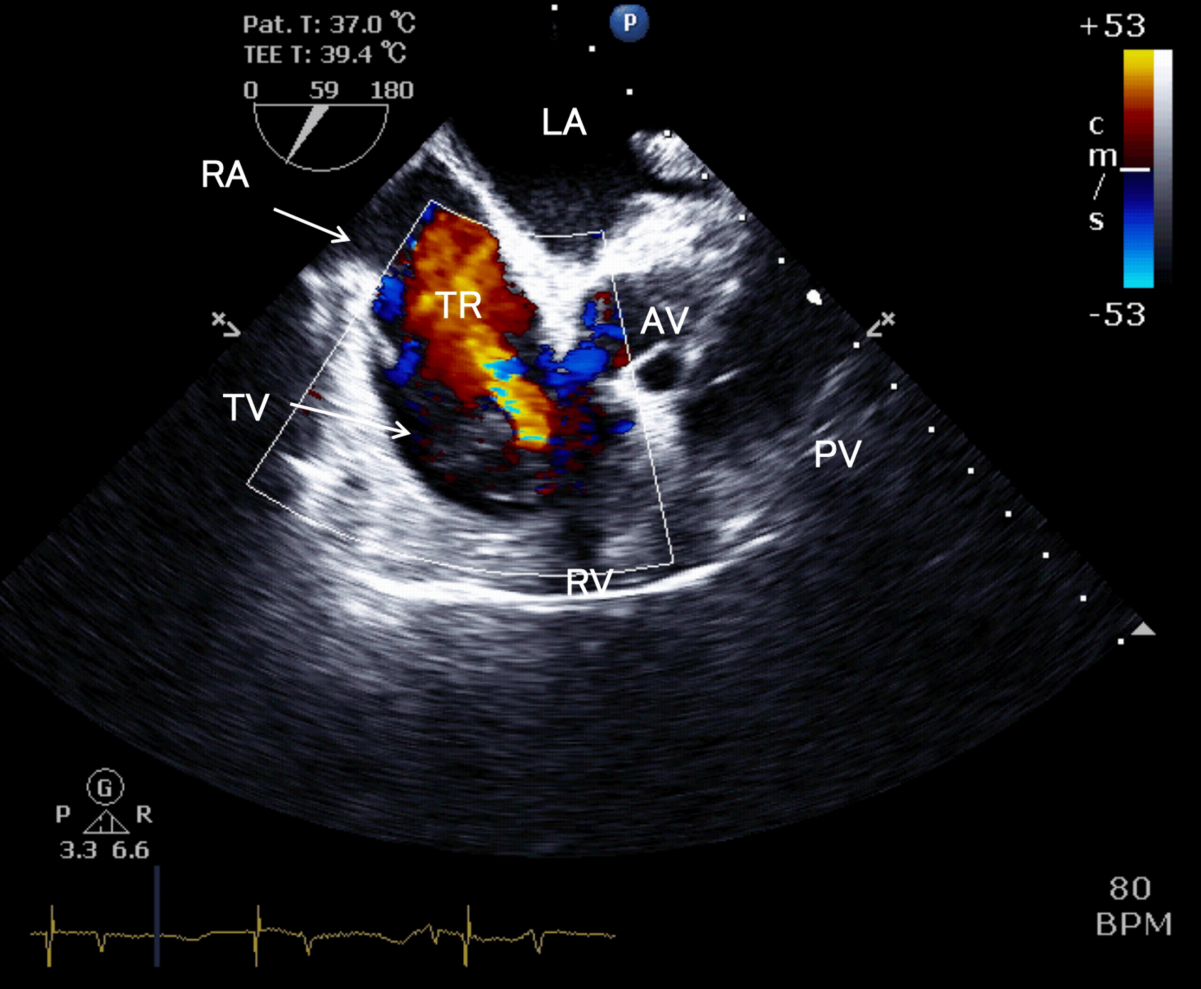
Post-procedural transesophageal echocardiography, right ventricular inflow-outflow view This is a right ventricle inflow-outflow view image taken after tumor resection and weaning from cardiopulmonary bypass. After removing the tumor that had impacted the tricuspid valve, moderate or severe tricuspid regurgitation was observed. Despite the tricuspid regurgitation, the right ventricular function remained good, unchanged from the preoperative state. TV, tricuspid valve; RA, right atrium; RV, right ventricle; TR, tricuspid regurgitation; LA, left atrium; AV, aortic valve; PV, pulmonary valve

**Figure 10:**
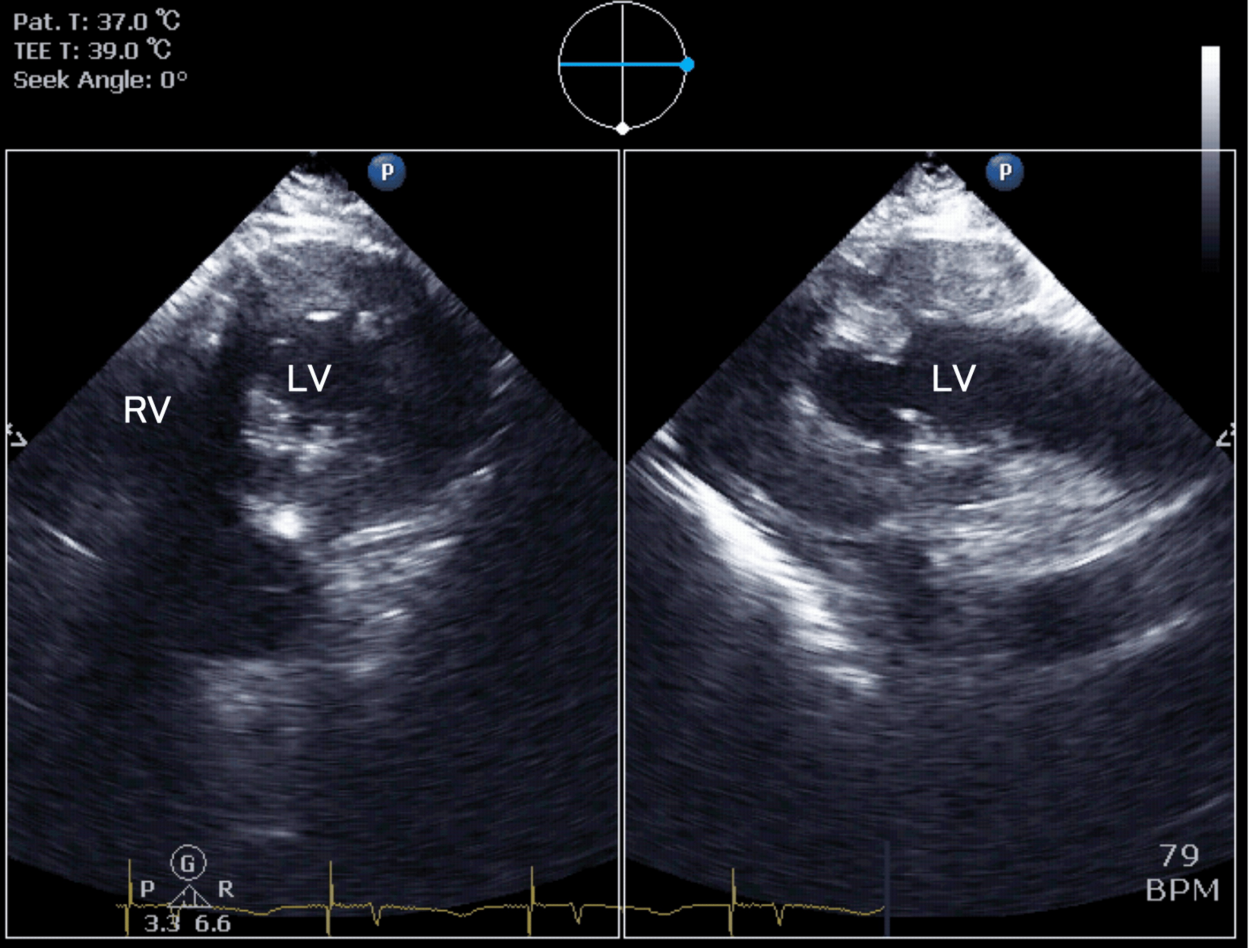
Post-procedural transesophageal echocardiography, transgastric bi-plane left ventricular view This image simultaneously bi-plane displays the short-axis and long-axis views of the left ventricle after tumor resection and weaning from cardiopulmonary bypass. The D-shaped deformity of the left ventricle that was observed preoperatively has improved. RV, right ventricle; LV, left ventricle

The TEE findings after tumor resection revealed that complete excision of the tumor from the IVC was not achieved, with residual tumor remaining. The right ventricular diameter continued to exceed that of the left ventricle. The left ventricular D-shaped deformity had resolved. Following the removal of the tumor that had been entrapped in the tricuspid valve, newly evident moderate to severe TR was observed (Figures 8-10).

After chest closure, the hemodynamics and respiratory function remained stable. Adequate hemostasis was achieved, and the blood products used during the surgery included 1200 mL of fresh frozen plasma and 400 mL of platelet concentrate. The drain output was low, and the patient was transferred to the ICU. The anesthesia time was 302 minutes, the surgery time was 225 minutes, the CPB time was 96 minutes, and the aortic cross-clamp and cardiac arrest time was 19 minutes.

The patient regained consciousness the following day; however, the cuff leak test was positive during the weaning assessment from mechanical ventilation. Due to concerns about post-extubation laryngeal edema, extubation was performed 56 hours after ICU admission, following the resolution of fluid overload with diuretics. She did not develop any new neurological abnormalities or require postoperative renal replacement therapy. She was discharged from the ICU on postoperative day five. Although the patient's general condition improved sufficiently to allow discharge two weeks postoperatively, due to the time required to coordinate complete resection of the residual tumor, the patient was temporarily discharged 30 days after the operation. The pathological diagnosis of the resected tumor was the smooth muscle tumor of uncertain malignant potential (STUMP), consistent with the histological findings of the uterine myoma 11 years prior.

## Discussion

The management of IVL with cardiac extension presents significant clinical challenges that require careful preoperative planning. Our case highlights essential considerations for the anesthetic management of advanced IVL with right heart obstruction. IVL is characterized by benign pelvic tumors, such as uterine leiomyomas, that invade the venous system, potentially reaching cardiac chambers. It predominantly affects pre-menopausal women with a mean age of onset around 45-50 years, with approximately 90% of patients having a history of uterine leiomyoma or prior hysterectomy [3]. The nonspecific clinical symptoms often include dyspnea, orthopnea, cough, syncope/presyncope, lower limb swelling, palpitations/tachycardia, and abdominal discomfort, with diagnosis frequently made incidentally after imaging studies [4,5]. Recurrence in the pelvis following surgical resection has been reported, with the recurrent tumor invading the vasculature [6].

This patient's presentation with circulatory collapse despite minimal previous symptoms reflects a typical pattern in IVL patients. The hormone-dependent nature of the tumor likely slowed its growth after menopause, allowing for the development of collateral circulation and compensatory mechanisms until the tumor became entrapped in the tricuspid valve, precipitating acute obstruction and shock. According to Wen et al.'s classification system, tumors can be categorized into four stages based on extension and three types based on adherence to vessel walls [7]. Their findings support CPB for tumor extraction in patients with stage 3 disease (extension into cardiac chambers) or higher, and type 2 (tumor adherent to vessel walls or endocardium) or type 3 (isolated tumor attached to vessel wall via pedicle) disease. In our case, the tumor's impaction in the tricuspid valve justified CPB implementation.

Regarding surgical approaches for IVL resection, some reports describe tumor extraction through the IVC via laparotomy without thoracotomy [8,9]. While CPB utilization carries disadvantages including increased systemic inflammatory response, potential tumor dissemination, and higher costs, Wen et al. also reported no significant differences in bleeding or complication rates between thoracotomy and laparotomy approaches in appropriate candidates [7]. Unlike aggressive malignancies such as renal cell carcinoma, IVL has a favorable prognosis after complete resection with a low risk of distant metastasis, supporting aggressive intervention when necessary. While VA-ECMO could be an alternative, CPB was preferred because our surgical approach required opening the right atrium for direct tumor visualization and excision. Technical challenges included obtaining adequate venous drainage with the IVC obstructed by the tumor. Despite being unable to advance the venous cannula near the right atrium, we could achieve approximately 70% of the target flow. It was essential to have alternative plans, such as additional SVC cannulation, in case a sufficient flow rate could not be achieved.

The anesthetic management of patients with right heart obstructive shock warrants special attention. Our strategy of establishing CPB before induction addressed several critical physiological concerns: loss of spontaneous ventilation following induction eliminates negative intrathoracic pressure that assists venous return; the patient's strong respiratory efforts with significant arterial pressure and CVP variations indicated heavy reliance on negative intrathoracic pressure; and elevated CVP with vasopressor dependence suggested impending circulatory collapse. A critical consideration for anesthesiologists is determining whether conventional induction is safe or extracorporeal circulation support is necessary before induction. Although clear criteria remain undefined, our case suggests that severe respiratory variations in hemodynamics, extreme elevation in CVP, and vasopressor requirements despite preserved consciousness should prompt consideration of establishing circulatory support before induction. When implementing pre-induction CPB, the careful combination of local anesthesia with analgosedation facilitated safe cannulation while minimizing risks of patient movement and excessive bleeding after heparinization.

## Conclusions

This case highlights a critical anesthetic consideration in managing intravenous leiomyomatosis with cardiac extension. In patients with severe respiratory variations in hemodynamics, elevated CVP, and vasopressor dependence despite preserved consciousness, establishing CPB before induction can be lifesaving. Our successful management demonstrates that with proper recognition of these physiological indicators and appropriate preoperative planning, even patients with advanced IVL and cardiovascular compromise can have excellent outcomes. As the literature on this topic is limited, further research will contribute to establishing the optimal management of this rare but potentially life-threatening condition.

## References

[1] Li B, Chen X, Chu YD, Li RY, Li WD, Ni YM (2013). Intracardiac leiomyomatosis: a comprehensive analysis of 194 cases. Interact Cardiovasc Thorac Surg.

[2] van der Heusen FJ, Stratmann G, Russell IA (2006). Right ventricular myxoma with partial right ventricular outflow tract obstruction. Anesth Analg.

[3] Ge Z, Wang Y, Wang Y, Li W, Yang X, Li J, Wang H (2023). Diagnostic experience of intravenous leiomyomatosis with emphasis on conventional ultrasonography imaging: a single-center study. Front Oncol.

[4] Clay TD, Dimitriou J, McNally OM, Russell PA, Newcomb AE, Wilson AM (2013). Intravenous leiomyomatosis with intracardiac extension - a review of diagnosis and management with an illustrative case. Surg Oncol.

[5] Xu ZF, Yong F, Chen YY, Pan AZ (2013). Uterine intravenous leiomyomatosis with cardiac extension: imaging characteristics and literature review. World J Clin Oncol.

[6] Worley MJ Jr, Aelion A, Caputo TA (2009). Intravenous leiomyomatosis with intracardiac extension: a single-institution experience. Am J Obstet Gynecol.

[7] Wen Y, Ma G, Miao Q (2025). The largest single-center report on intravenous leiomyomatosis and development of a classification to guide surgical management. J Vasc Surg Venous Lymphat Disord.

[8] Harris LM, Karakousis CP (2000). Intravenous leiomyomatosis with cardiac extension: tumor thrombectomy through an abdominal approach. J Vasc Surg.

[9] Rispoli P, Santovito D, Tallia C, Varetto G, Conforti M, Rinaldi M (2010). A one-stage approach to the treatment of intravenous leiomyomatosis extending to the right heart. J Vasc Surg.

